# Increasing the Gas Barrier Properties of Polyethylene Foils by Coating with Poly(methyl acrylate)-Grafted Montmorillonite Nanosheets

**DOI:** 10.3390/polym13193228

**Published:** 2021-09-23

**Authors:** Judith E. Rauschendorfer, Philipp Vana

**Affiliations:** Institute of Physical Chemistry, Georg-August-University Göttingen, Tammannstraße 6, 37077 Göttingen, Germany; jrauschen@chemie.uni-goettingen.de

**Keywords:** montmorillonite nanosheets, RAFT polymerization, gas barrier properties, coating, surface-grafted polymer, LDPE foils, food packaging

## Abstract

Low-density polyethylene (LDPE) foils were coated with a thin film of polymer-grafted Montmorillonite (MMT) nanosheets, which form a barrier against gas diffusion due to their unique brick-and-mortar arrangement. The MMT nanosheets were grafted with poly(methyl acrylate) (PMA), a soft and flexible polymer. Already very thin films of this nanocomposite could reduce gas permeability significantly. The impact of the topology of the surface-grafted polymer on gas permeability was also studied. It was found that grafting MMT nanosheets with a mixture of star-shaped and linear PMA and with PMA that is cross-linked via hydrogen bonds further decrease gas permeability. The presented strategy is quick and simple and allows for the easy formation of effective gas barrier coatings for LDPE foils, as used in food packaging.

## 1. Introduction

Multilayer systems are omnipresent in modern high-performance materials. For example, polymer-based food packaging is a highly sophisticated layer system with usually at least one layer providing a barrier against gas diffusion [1]. One of the most fundamental components for this layered system is low-density polyethylene (LDPE) which is one of the most commonly used technical commodity polymers [1]. While LDPE provides excellent barrier properties against moisture, it only shows poor barrier characteristics against oxygen, flavour, and aroma, all of which are necessary to keep the product fresh and the consumers safe [1,2]. Additional barrier layers are thus applied in order to enhance the materials performance. Known ways to improve the barrier properties of foils are, for example, the use of additional layers of proteins [3,4], copolymers [5], or nanoparticles [6].

Montmorillonite (MMT) is a naturally occurring layered silicate that has been shown to provide an exceptional barrier against the diffusion of gasses, when used as filler for polymers [1,2,5]. Its presence in the polymer matrix results in the formation of a “tortuous” path for diffusion increasing the mean path length that gas molecules have to travel to pass the material [6,7]. Commonly, MMT nanosheets are blended directly into the polymer matrix [7,8,9,10] with all the problems of aggregation and cluster-formation that effectively hampers the complete unfolding of their beneficial properties. However, when arranged tightly in a “brick-and-mortar” structure [11,12] as a separate layer or coating, the packing density and with that also the mean path length for diffusion of gas molecules can be significantly increased [2,13]. As “mortar”, synthetic polymers can be used and f they are synthesized via controlled polymerization techniques, e.g., by reversible addition-fragmentation chain transfer (RAFT) polymerization, their properties can be precisely tailored [14,15]. They do not only provide stability to the MMT layer but can also be used to ensure sufficient interaction between different layers.

We have recently presented a reliable strategy to graft linear poly(methyl acrylate) (PMA) to MMT nanosheets via a surface-confined grafting-through RAFT polymerization [14,15]. The resulting exfoliated and polymer-covered MMT nanosheets were employed as advanced filler material that is capable of tuning the mechanical properties of PMA over a wide range. In addition, self-standing films of matrix-free polymer-covered MMT nanosheets were developed that may imitate the microscopic structure of nacre and show improved mechanical properties. We also presented in these studies how the topology of the grafted PMA can be altered from linear to a mixture of star-shaped and linear PMA and how this strategy can be used to further optimize the mechanical properties of the nanocomposites. Additionally, we showed that by addition of a hydrogen-bonding comonomer to the grafted linear PMA the properties of the composites can be further enhanced [14]. We demonstrated that these changes in the grafted polymer allow for an increase in mechanical stability of the arranged polymer/nanosheet structure. As this structure is also crucial for the formation of a gas barrier, these novel matrix-free hybrid nanosheets are now adapted to form coatings for LDPE foils and are for the first time analyzed for their gas barrier properties. The polymer-grafted MMT nanosheets—without any additional matrix polymer—are cast to the surface of LDPE forming a µm-thin coating, which effectively decreases gas diffusion.

## 2. Materials and Methods

### 2.1. Materials

2-(Dodecylthiocarbonothioylthio)propionic acid (CTA, Sigma Aldrich, St. Louis, Missouri, USA) was used without further purification. Azobis(isobutyronitril) (AIBN, ≥99%, Fluka, St. Louis, MI, USA) was recrystallized from methanol and stored at −18 °C. Methyl acrylate (MA, Sigma Aldrich, St. Louis, MI, USA) and 2-carboxyethyl acrylate (CEA, Sigma Aldrich, St. Louis, MI, USA) were purified by passing through a column of basic alumina (Sigma Aldrich, St. Louis, MI, USA) to remove the inhibitor. Sodium MMT was purchased from Alfa Aesar, Kandel, Germany. Propylen glycol monomethyl ether acetate (PGMEA, p.a., J&K Scientific, Beijing, China) was used as received. The used three-arm star shaped RAFT agent S3 was synthesized following a literature known procedure [16].

### 2.2. Synthesis of Polymer-Grafted MMT Nanosheets

MMT nanosheets grafted with linear PMA, a mixture of linear and star-shaped PMA, and linear PMA with the hydrogen bonding comonomer CEA were prepared according to the protocol published recently by our group [14,15]. Therefore, MMT was grafted with an ionic monomer (VB16) via ion exchange as presented by Salem and Shipp [17]. The VB16 grafted MMT nanosheets (MMT-VB16) were then modified with polymer in a grafting-through RAFT polymerization using CTA as RAFT agent to produce a linear polymer or a combination of CTA and a three-arm star shaped RAFT (S3) agent to yield a partially star shaped polymer: MMT-VB16 (0.196 g) was weighted into a polymerization vial equipped with a magnetic stirrer bar. MA (2.850 g, 0.331 mol), AIBN (2.7 mg, 1.66·10^−^^6^ mol) and the respective RAFT agent (CTA: 29.0 mg, 8.28·10^−5^ mol or a mixture CTA(14.5 mg, 4.14·10^−5^ mol)/S3(26.6 mg, 4.16·10^−5^ mol)), and toluene (4.275 g) were added. To obtain poly(MA-co-CEA) modified nanosheets, analogue quantities of MA and AIBN were used. CTA (29.0 mg, 8.28·10^−^^5^ mol) and CEA (97.7 mg, 6.78·10^−4^ mol) were added. All vials were degassed with argon for 15 min and then polymerized at 60 °C for 3 h under constant stirring to yield polymer modified MMT nanosheets. The polymerizations were stopped by exposure to air and dilution with cooled dichloromethane. Free polymer was washed away by three cycles of centrifugation (25 °C, 30 min, 9000 rpm, 8603 g) followed by redispersion in dichloromethane.

### 2.3. Coating of LDPE Foils with Polymer Grafted Nanosheets

PMA-grafted MMT nanosheets were dispersed in propylene glycol methyl ether acetate (PGMEA) (5 wt%). LDPE foils (SiBo, Wenden, Germany, thickness: 0.05 mm) were dip-coated with an automated dip-coater into the dispersion and then hung up to dry at room-temperature for 24 h. The weight fraction of the coating was determined gravimetrically by comparing the weight before and after coating.

### 2.4. Methods

#### 2.4.1. Gas Permeability Measurements

**Figure 1:**
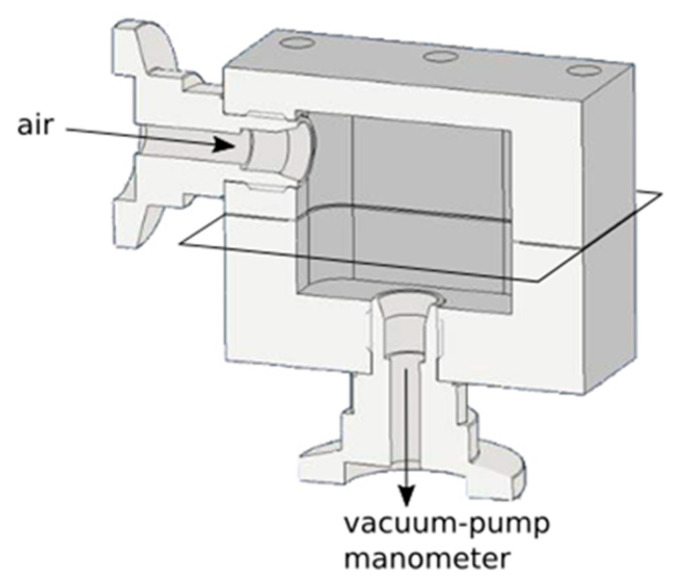
Experimental setup for gas permeability measurements. A foil (black outlines) is placed between two hollow metal blocks. One side (top) is open to the environment, the other (bottom) is connected to a vacuum pump and a manometer. The pump can be sealed off with a valve. Gas barrier properties are determined by reducing the pressure on the bottom side to 400 mbar and then recording the rise in pressure time-dependently.

The gas permeability measurements were conducted using a self-made setup shown in Figure 1 using air. The setup was equipped with a vacuum pump (LVS 210, Welch-Ilmvac, Fürstenfeldbruck, Germany) and a manometer (Vacuubrand GmbH, Wertheim, Germany). The pressure was reduced to 400 mbar and the time-dependent change in pressure was recorded.

#### 2.4.2. Thermogravimetric Analysis

Thermogravimetric analysis (TGA) was used to determine the polymer content of the nanosheets used for the coatings. It was performed with a Netzsch (Selb, Germany) TG 209 F3 Tarsus over a temperature range from room temperature to 1000 °C with a heating rate of 10 K min^−^^1^ under nitrogen atmosphere using Al_2_O_3_ crucibles that were pyrolytically cleaned at 1000 °C prior to each measurement.

#### 2.4.3. NMR

^1^H-NMR was used to determine the CEA content of the MMT-PMA nanosheets. Spectra were measured with a Varian Unity 300 at room temperature. As solvent dichloromethane-d2 was used. The signal obtained from residual solvent protons was used as an internal standard for all measurements. All spectra were base-line corrected with MestreNova v10.0.2-15465 (Mestrelab Research SL, Santiago de Compostela, Spain).

## 3. Results

### 3.1. Sample Preparation

To analyze the impact of the PMA nanosheet coatings on the gas barrier properties of LDPE, foils (approx. thickness of 0.5 mm) were dip-coated in dispersions of PMA-grafted MMT nanosheets in PGMEA, which is a good solvent for the grafted polymer chains, allowing entanglement between chains grafted to different nanosheets, but is a poor solvent for LDPE. The special situation of these PMA-grafted MMT nanosheets is that there is no free matrix-polymer present and necessary to form stable films, which inherently leads (i) to a very high MMT/polymer ratio and (ii) to very evenly dispersed MMT nanosheets in the final material. It is this matrix-free situation that makes this material beneficial for this targeted application. The coated foils were then left to dry for 24 h at room temperature. Three different coatings were applied and compared: nanosheets grafted with (a) linear PMA (MMT-PMA_L_), (b) a mixture of star and linear PMA (MMT-PMA_S_) and (c) with linear PMA that contained 2.6 mol% of carboxyethyl acrylate (CEA), a hydrogen bonding comonomer (MMT-PMA_HB_). The CEA content after the statistical copolymerization of CEA and MA was determined using NMR. We demonstrated in an earlier publication that a low CEA content is already sufficient to achieve a significant impact on the mechanical performance of the material, while a content that is too high would result in the material turning brittle [14]. Via thermogravimetric analysis (m), it was guaranteed that all polymer-covered MMT nanosheets were grafted with exactly the same amount of polymer (65 wt%) in order to ensure that differences observed in the gas permeability measurements can be attributed only to structural differences of the three different kinds of particles, but not to a different ratio of polymer to nanosheets in the coating. To analyze the influence of the coating’s thickness, foils were coated either once or three times. The weight fraction of the coating was analyzed gravimetrically and only foils with exactly the same amount of coating, thus with exactly the same coating thickness, were compared. For LDPE foils that were coated once, the fraction of the coating was 0.79 ± 0.02 wt% and for samples coated three times the coating fraction was 2.77 ± 0.13 wt%. Measuring the exact film thickness of a polymer-containing coating covering a polymer film as substrate is, experimentally, extremely demanding and was thus not performed in this work, as this information was not needed for this study. Using dip coating, however, can lead to coating thicknesses in the order of several tens of µm, mainly depending on solution viscosity and withdrawal speed [18].

### 3.2. Analysis of the Gas Barrier Properties

The barrier properties of the MMT-PMA coatings were analyzed using the in-house-designed setup shown in Figure 1. A coated polymer film sample is tightly screwed between two hollow metal blocks. The top part has an opening where gas from the environment can enter freely. The bottom part is attached to a manometer and a vacuum pump that can be tightly closed via a valve. When the foil is loaded into the setup, the pressure on the bottom side is reduced to 400 mbar. Then the valve between sample and pump is closed and the rising pressure is recorded over time.

**Figure 2:**
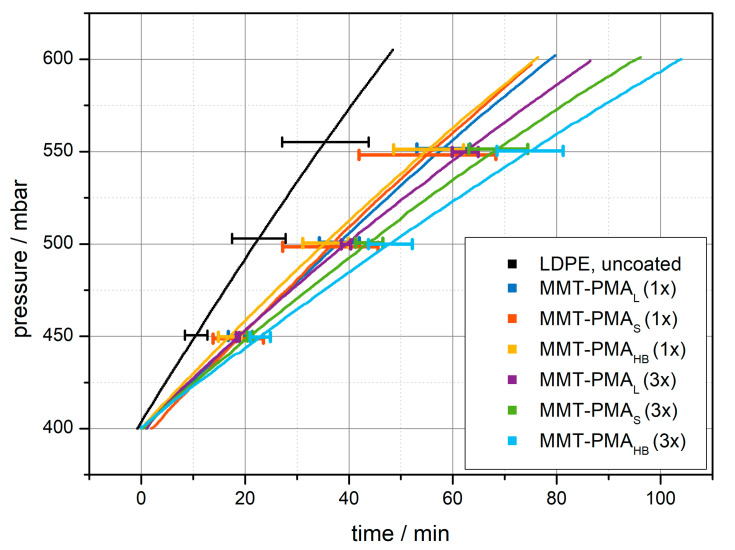
Pressure-time-profiles of LDPE foils coated with MMT-PMA_L_, MMT-PMA_S_, or MMT-PMA_HB_. Numbers in brackets indicate the number of coating cycles. For visual clarity, only three error bars per curve are shown. Errors are determined as the standard deviation from at least three measurements.

From these experiments, pressure–time-profiles can be recorded (Figure 2). The longer it takes for the pressure to reach 600 mbar, the lower the gas permeability of the sample and the higher the sample’s gas barrier properties. Additionally, Table 1 shows the obtained average durations Δ*t*_400__→__600mbar_ for all tested samples. Δ*t*_400__→__600mbar_ corresponded to the measured duration until the pressure in the cell had risen from 400 mbar to 600 mbar. It can be seen that all coated foils show a significantly lower gas permeability than the uncoated LDPE foil. Foils coated once all show a similar gas permeability reduction independently of the polymer-modification of the nanosheets. This may be due to the fact, that the coating is relatively thin and the different modifications of the nanosheets has not yet resulted in a difference in the packing density of the nanosheets. Coating the foils three times demonstrates the thickness dependency of the gas barrier properties, as Δ*t*_400__→__600mbar_ was found to be significantly increased. Additionally, a trend of increasing barrier properties from coating with MMT-PMA_L_ over MMT-PMA_S_ to MMT-PMA_HB_ could be observed. A possible interpretation might be that by cross-linking the polymer, a denser and more compact coating is obtained. In the MMT-PMA_S_ sample, cross-linking occurs via the central branching-points of incorporated star polymers and in MMT-PMA_HB_ cross-linking appears via hydrogen-bonds. Both situations may result in a denser coating, where the mean path length of gas molecules diffusing through the material is increased, which can be observed as increased barrier properties or reduced gas permeability, respectively.

## 4. Conclusions

LDPE foils were coated with polymer grafted MMT nanosheets to demonstrate their increased barrier properties against gas diffusion. The impact of the coating’s thickness was studied through variation of the number of coating cycles. It was found that the gas permeability can be controlled through the coating’s thickness. Additionally, the impact of the polymer’s topology and physical properties was investigated. The nanosheets were coated with either linear PMA, a mixture of star-shaped and linear PMA, or PMA that was cross-linked through the addition of hydrogen bonding sites within a small proportion of the monomer units. It was demonstrated that the coatings of nanosheets grafted with cross-linked PMA exhibited higher gas barrier properties than coatings made from nanosheets grafted with linear PMA.

## Figures and Tables

**Table 1:** Average durations Δt_400__→__600mbar_—the time difference between 400 and 600 mbar in the sample chamber—for LDPE-foils coated with MMT nanosheets modified with PMA_L_, PMA_S_ or PMA_HB_. Errors are obtained as the standard deviation of at least three measurements.

Sample	Number of Coating Cycles	Δ*t*_400__→__600mbar_/min
LDPE	0	38 ± 11
LDPE + MMT-PMA_L_	1	79 ± 11
LDPE + MMT-PMA_S_	1	74 ± 16
LDPE + MMT-PMA_HB_	1	77 ± 8
LDPE + MMT-PMA_L_	3	86 ± 4
LDPE + MMT-PMA_S_	3	96 ± 8
LDPE + MMT-PMA_HB_	3	104 ± 8

## Data Availability

The data presented in this study are available on request from the corresponding author.

## References

[1] Anukiruthika T., Sethupathy P., Wilson A., Kashampur K., Moses J.A., Anandharamakrishnan C. (2020). Multilayer packaging: Advances in preparation techniques and emerging food applications. Compr. Rev. Food Sci. Food Saf..

[2] Ding F., Liu J., Zeng S., Xia Y., Wells K.M., Nieh M.-P., Sun L. (2017). Biomimetic nanocoatings with exceptional mechanical, barrier, and flame-retardant properties from large-scale one-step coassembly. Sci. Adv..

[3] Schmid M., Dallmann K., Bugnicourt E., Cordoni D., Wild F., Lazzeri A., Noller K. (2012). Properties of whey-protein-coated films and laminates as novel recyclable food packaging materials with excellent barrier properties. Int. J. Polym. Sci..

[4] Cinelli P., Schmid M., Bugnicourt E., Wildner J., Bazzichi A., Anguillesi I., Lazzeri A. (2014). Whey protein layer applied on biodegradable packaging film to improve barrier properties while maintaining biodegradability. Polym. Degrad. Stab..

[5] Topolniak I., Gardette J.L., Therias S. (2015). Influence of zeolite nanoparticles on photostability of ethylene vinyl alcohol copolymer (EVOH). Polym. Degrad. Stab..

[6] Wolf C., Angellier-Coussy H., Gontard N., Doghieri F., Guillard V. (2018). How the shape of fillers affects the barrier properties of polymer/non-porous particles nanocomposites: A review. J. Memb. Sci..

[7] Vilarinho F., Vaz M.F., Silva A.S. (2019). The Use of Montmorillonite (MMT) in Food Nanocomposites: Methods of Incorporation, Characterization of MMT/Polymer Nanocomposites and Main Consequences in the Properties. Recent Pat. Food. Nutr. Agric..

[8] Majeed K., Arjmandi R., Hassan A. (2018). Mechanical and oxygen barrier properties of LDPE/MMT/MAPE and LDPE/MMT/EVA nanocomposite films: A comparison study. J. Phys. Sci..

[9] Xie L., Lv X.Y., Han Z.J., Ci J.H., Fang C.Q., Ren P.G. (2012). Preparation and Performance of High-Barrier Low Density Polyethylene/Organic Montmorillonite Nanocomposite. Polym.-Plast. Technol. Eng..

[10] Mooninta S., Poompradub S., Prasassarakich P. (2020). Packaging Film of PP/LDPE/PLA/Clay Composite: Physical, Barrier and Degradable Properties. J. Polym. Environ..

[11] Chen R., Wang C., Huang Y., Le H. (2008). An efficient biomimetic process for fabrication of artificial nacre with ordered-nanostructure. Mater. Sci. Eng. C.

[12] Liang B., Zhao H., Zhang Q., Fan Y., Yue Y., Yin P., Guo L. (2016). Ca 2+ Enhanced Nacre-Inspired Montmorillonite–Alginate Film with Superior Mechanical, Transparent, Fire Retardancy, and Shape Memory Properties. ACS Appl. Mater. Interfaces.

[13] Priolo M.A., Holder K.M., Guin T., Grunlan J.C. (2015). Recent advances in gas barrier thin films via layer-by-layer assembly of polymers and platelets. Macromol. Rapid Commun..

[14] Rauschendorfer J.E., Möckelmann J., Vana P. (2021). Enhancing the Mechanical Properties of Matrix-Free Poly(Methyl Acrylate)-Grafted Montmorillonite Nanosheets by Introducing Star Polymers and Hydrogen Bonding Moieties. Macromol. Mater. Eng..

[15] Rauschendorfer J.E., Thien K.M., Denz M., Köster S., Ehlers F., Vana P. (2020). Tuning the Mechanical Properties of Poly(Methyl Acrylate) via Surface-Functionalized Montmorillonite Nanosheets. Macromol. Mater. Eng..

[16] Rossner C., Tang Q., Glatter O., Müller M., Vana P. (2017). Uniform Distance Scaling Behavior of Planet-Satellite Nanostructures Made by Star Polymers. Langmuir.

[17] Salem N., Shipp D.A. (2005). Polymer-layered silicate nanocomposites prepared through in situ reversible addition-fragmentation chain transfer (RAFT) polymerization. Polymer.

[18] Scriven L.E. (1988). Physics and Applications of DIP Coating and Spin Coating. MRS Proc..

